# Agriculture’s impact on water–energy balance varies across climates

**DOI:** 10.1073/pnas.2410521122

**Published:** 2025-03-17

**Authors:** Masoud Zaerpour, Shadi Hatami, André S. Ballarin, Simon Michael Papalexiou, Alain Pietroniro, Ali Nazemi

**Affiliations:** ^a^Department of Civil Engineering, Schulich School of Engineering, University of Calgary, Calgary, AB T2N 1N4, Canada; ^b^Department of Hydraulics and Sanitation, São Carlos School of Engineering, University of São Paulo, São Paulo 13566-590, Brazil; ^c^Department of Water Resources and Environmental Modeling, Faculty of Environmental Sciences, Czech University of Life Sciences, Prague 165 00, Czech Republic; ^d^Department of Building, Civil, and Environmental Engineering, Concordia University, Montreal, QC H3G 2W1, Canada

**Keywords:** agriculture, water balance, irrigation, Budyko water balance

## Abstract

Agriculture is a cornerstone of global food production, accounting for a substantial portion of water withdrawals worldwide. As the world’s population grows, so does the demand for water in agriculture, leading to alterations in regional water–energy balances. We present an approach to identify the influence of agriculture on the water–energy balance using empirical data. We explore the departure from the Budyko curve for catchments with agricultural expansion and their associations with changes in the water–energy balance using a causal discovery algorithm. Analyzing data from 1,342 catchments across three Köppen-Geiger climate classes—temperate, snowy, and others—from 1980 to 2014, we show that temperate and snowy catchments, which account for over 90% of stations, exhibit distinct patterns. Cropland percentage (CL%) emerges as the dominant factor, explaining 47 and 37% of the variance in deviations from the Budyko curve in temperate and snowy catchments, respectively. In temperate catchments, CL% shows a strong negative correlation with precipitation-streamflow (P-Q) causal strength (Spearman ρ=−0.75), suggesting that cropland exacerbates precipitation-driven deviations. A moderate negative correlation with aridity-streamflow (AR-Q) causal strength (ρ=−0.42) indicates additional influences of cropland through aridity-driven interactions. In snowy catchments, CL% is similarly influential, with a positive correlation with P-Q causal strength (ρ=0.51). However, the negative correlation with AR-Q causal strength (ρ=−0.45) underscores the role of aridity as a secondary driver. While vegetation and precipitation seasonality also contribute to the deviations, their impacts are comparatively lower. These findings underscore the need for inclusion of agricultural activities in changing water–energy balance to secure future water supplies.

## Significance

Agriculture plays a key role in global food security, intricately tied to water resources for crop growth. However, navigating the interplay between agriculture and water availability poses challenges, especially during the *Anthropocene*, where traditional perspectives often overlook agriculture’s impacts on the water cycle. Understanding and integrating agriculture’s influence on water dynamics becomes imperative in addressing contemporary challenges. Our study highlights the contrasting impacts of agricultural activities across temperate and snowy climates. In temperate catchments, agriculture weakens the precipitation-streamflow (P-Q) relationship, contributing to precipitation-driven deviations from the water–energy balance, while in snowy catchments, agricultural activities strengthen the P-Q relationship. These findings offer insights for shaping effective water management strategies, ensuring food security, and promoting sustainable development globally.

## 1.1. Context.

Agriculture is crucial for meeting the world’s growing food demands and accounts for approximately 72% of global water withdrawals (1–4). Global estimates of water utilized for crops span from 2,217 to 3,185 km^3^ per year (5–8), while additional crop evapotranspiration encompasses a range of 927 to 1,530 km^3^ per year (6, 8, 9). As the global population continues to increase, the need for water in agriculture has surged, resulting in often unknown consequences for the hydrological cycle. To sustain increasing food demand resulting from population growth and elevated living standards, global water withdrawals have surged nearly sixfold, escalating from approximately 500 km^3^ per year in 1,900 to nearly 3,000 km^3^ per year in 2000. During this period, crop yields have also substantially increased, reflecting significant advances in agricultural productivity (10); however, improved water use efficiency does not necessarily reduce total water consumption (11). Consequently, agriculture remains a predominant water user globally (8, 12, 13). While agricultural expansion sustains food security, at the same time, it has triggered severe water scarcity issues at regional to global levels (14, 15). For instance, the agriculture region in and around the Ogallala Aquifer, located in the Great Plains region of the United States, witnessed a dramatic increase in agricultural area, surging from 8,500 km^2^ in 1949 to 63,000 km^2^ by 2005 (16). Sustaining such a level of agricultural productivity, however, hinged on the ongoing extraction of water from the Ogallala Aquifer (17, 18).

Crop water needs can be fulfilled through three distinct sources: (1) green water, derived from local precipitation and temporarily stored in the soil, (2) blue water, encompassing surface water from local/nonlocal rivers, lakes, reservoirs, and renewable groundwater, and (3) nonrenewable groundwater, extracted from aquifers (19, 20). The term “nonrenewable groundwater abstraction” is used to underscore water extracted beyond recharge levels for a prolonged period, i.e., physically unsustainable groundwater use (21) that is unlikely to be replenished in years (22, 23).

Surface water is the primary source of freshwater for human use, with around 70% of the available surface resource used in agriculture (24). However, blue water alone does not fully represent the water needed for crop production (25–27), as rainfed crops primarily rely on rainfall infiltration, which constitutes a significant portion of water for agricultural croplands (28, 29). Additionally, actual agricultural water consumption is better measured as the difference between crop water use and what would be required by the natural vegetation that was replaced (30). As surface water sources become less reliable and predictable (31, 32), the importance of groundwater for agriculture is on the rise. In regions with limited access to surface water, groundwater can emerge as the primary source of agricultural water (33). However, unsustainable groundwater depletion can easily arise when extraction surpasses recharge over prolonged periods, resulting in declining groundwater levels and consequently reduced baseflow (20, 23, 33–39). Groundwater recovery can be gradual, with residence times spanning from several months in shallow aquifers to millions of years in deep aquifers. Hence, it is crucial to recognize unsustainable agricultural practices and their far-reaching consequences in altering the hydrological cycle.

A comprehensive understanding of the water cycle enhances our evaluation of freshwater resources and enables us to understand global water security threats more effectively. Also, on a global scale, climate change has been identified as a primary driver of hydrological cycle changes (40–42). Nonetheless, the hydrological cycle is not solely shaped by climate dynamics (43); it is also influenced by human activities like agriculture and groundwater extraction at the regional scale (44–46). Understanding the agricultural practices by source and their interplay with other drivers of changes in streamflow is imperative for advancing hydrological science at regional and global scales. For instance, surface water plays a pivotal role in flood dynamics (47–49), whereas groundwater is closely tied to baseflow changes and bears immense significance for environmental flow management and water supply in arid and semiarid regions (50–52). Two notable examples of groundwater use for agriculture in the United States are the Ogallala aquifer in High Plains and the aquifer system in California’s Central Valley. These aquifers are extensively studied, with depletion estimates drawn from numerous well water level measurements and GRACE satellite data (36, 53, 54).

## 1.2. Combining the Budyko Curve with Causal Analysis Is a Way to Provide Insight Into How Agriculture Affects the Water Cycle.

Despite the important role of agriculture and other anthropogenic water uses in shaping the hydrological cycle, assessing their global impact remains challenging due to uncertainties associated with factors such as agricultural area delineation, hydrological modeling, and critical local parameters such as soil hydraulic characteristics, the timing of the growing season, and its relation to water availability (3, 55).

By applying the Budyko water balance framework (56) and a causal discovery algorithm (57, 58), we explore the effect of agricultural water use on the hydrological cycle. The interplay of components of terrestrial freshwater ecosystems is uncertain in an era of human modifications and rapid global climate change. Although previous methods employed modeled streamflow data to identify causal connections between human-induced forcing and observed river flow patterns globally, uncertainties remain regarding the complex interactions between human activity, climate variables, and catchment-scale hydrological processes that impact water flow dynamics. Building on previous findings, we have used a large, measured dataset to better understand watershed responses to human changes in land surface caused by agricultural expansion.

The Budyko hypothesis offers a valuable tool for comparing normalized observations across a wide spectrum of climatic conditions, enabling the identification of secondary controls on a catchment’s water balance (45, 59, 60). According to the Budyko curve, the ratio of mean annual evapotranspiration to mean annual precipitation (E/P, the evaporative fraction) is primarily influenced by the ratio of mean annual potential evapotranspiration to mean annual precipitation (Ep/P, the dryness index). Hereafter, the term water–energy balance refers to the streamflow-precipitation-aridity equilibrium described by the Budyko curve. Using this framework, we study the impact of agriculture by source on the water–energy balance at the country scale. Additionally, it is important to consider the role of precipitation seasonality and vegetation in influencing deviations from the Budyko curve. Studies have shown that seasonality of precipitation and potential evapotranspiration can substantially alter the timing and efficiency of water inputs to ecosystems, affecting evapotranspiration and runoff patterns (61, 62). Variations in vegetation type and density can further complicate these interactions, as they influence the partitioning of water between evapotranspiration and runoff (63, 64). Understanding these relationships is crucial for accurately interpreting the Budyko curve and the hydrological responses of different catchments.

It is essential to acknowledge that the Budyko curve, while highly informative, does not represent the complexity of natural hydrological processes. However, the Budyko curve follows a well-known characteristic curve when estimated for a series of natural basins. The spatial differences in the water balance between catchments can result from various factors beyond just agricultural water use. In addition, it is essential to acknowledge that a direct symmetry might not always exist in the temporal partitioning of precipitation into streamflow and evaporation within a catchment, in contrast to the observed spatial variations among catchments (65–67).

Because of these limitations, we also analyzed the interplay between main components of water–energy balance and their relationship with the cropland percentage (CL%; as an agriculture proxy), thus shedding light on the effect of agriculture on altering water–energy balance. Many Budyko studies often focus solely on spatial patterns of long-term averages (45, 66, 68, 69). Nevertheless, this methodology frequently omits a substantial amount of hydrological information and the interplay of various factors that could influence water balance at finer temporal scales (68). To address this challenge and gain insight into the intricate interplay of factors at finer time scales, we analyzed the control of agriculture on temporal changes of individual responses at the seasonal time scale (67, 69) using the PCMCI+ causal discovery method recently developed and applied in Earth system sciences (57, 58). By integrating the Budyko framework with the PCMCI+ causal discovery algorithm, we offer an approach to quantifying the equilibrium between water and energy balance in catchments. Our examination focuses on three key factors influencing streamflow (Q): precipitation (P), aridity (AR), and snow fraction (SF) (67, 70, 71). For calculating the SF, we assumed that precipitation falls as snow when the temperature is below 1 °C (see the *Materials and Methods* for further information on calculating the SF). We establish causal relationships between these drivers and streamflow and measure their strengths using the causal discovery method (58).

Here, we study the role of agriculture by source in altering the water–energy balance using 1,342 Catchment Attributes and Meteorology for Large-sample Studies (CAMELS) catchments located across the contiguous United States and Great Britain (GB) from 1980 to 2014. The role of agriculture is studied by a spatial (between-catchment) comparison of the long-term partitioning of precipitation into evapotranspiration and streamflow due to the lack of CL% time series. These long-term observations are then conceptualized within the Budyko framework (56).

## 2. Results

**Fig. 1.**
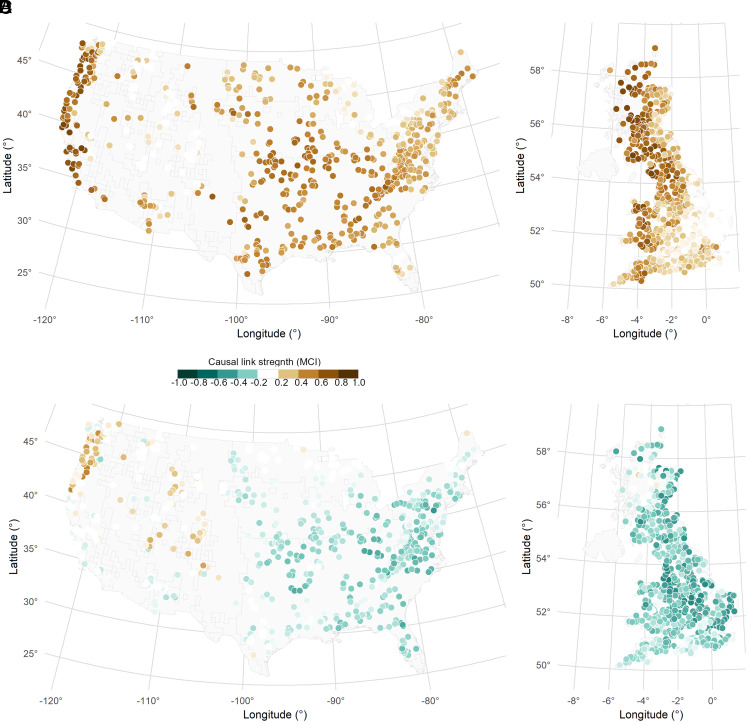
Quantifying the causal link strength between main components of water–energy balance including streamflow, precipitation, and AR. The strength of these causal links is assessed through the MCI test statistic, where MCI values range from –1 to 1. Panels (*A*) and (*B*) illustrate the strength of causal links between streamflow-precipitation (P-Q) in the United States and Great Britain (GB), respectively. Panels (*C*) and (*D*) depict the strength of causal links between streamflow-aridity (AR-Q) in the United States and GB, respectively. White dots indicate catchments where the causal link strengths are not statistically significant.

We first evaluate the causal strength between main components of water–energy balance including streamflow, precipitation, and AR, as depicted in Fig. 1. This analysis provides insights into the water–energy dynamics from the causal perspective. Causal link strength is quantified based on the momentary conditional independence (MCI) test statistic value (57, 58, 72, 73). The MCI test statistics yield a well-interpretable notion of a normalized causal strength (MCI test statistics value) that allows to measure the strength of causal links between variables (74, 75). An MCI value of +1 denotes the strongest positive link, while -1 represents the strongest link with a negative impact. We acknowledge that the hydrological components are inherently interconnected through the principle of mass conservation, and deviations in one component are necessarily reflected in another. However, our analysis focuses on the temporal interactions and perturbations that drive catchments toward particular long-term balances, including deviations from the Budyko curve. These deviations are not a violation of mass balance but rather an indication of how external factors influence the partitioning and distribution of water–energy components.

Panels (*A*) and (*C*) of Fig. 1 present the results for precipitation-streamflow (P-Q) and aridity-streamflow (AR-Q) links in the United States, respectively, while Panels (*B*) and (*D*) showcase corresponding results for GB. In the United States, precipitation emerges as the predominant factor influencing streamflow, demonstrating an expected MCI value of 0.43. AR also exerts control over streamflow, particularly in catchments located in Central and Coastal Plains; however, the expected MCI value of −0.12 for all catchments in the United States is not statistically significant. In GB, similar to the US findings, precipitation predominantly control streamflow, with an expected MCI value of 0.41. AR also plays a significant role in streamflow, particularly evident in East England, with an expected MCI value stands at −0.31 across all catchments. The results related to SF-Q can be found in *SI Appendix*, Fig. S1.

### 2.1. The Percentage of Cropland May Explain Deviations from the Budyko Curve.

**Fig. 2.**
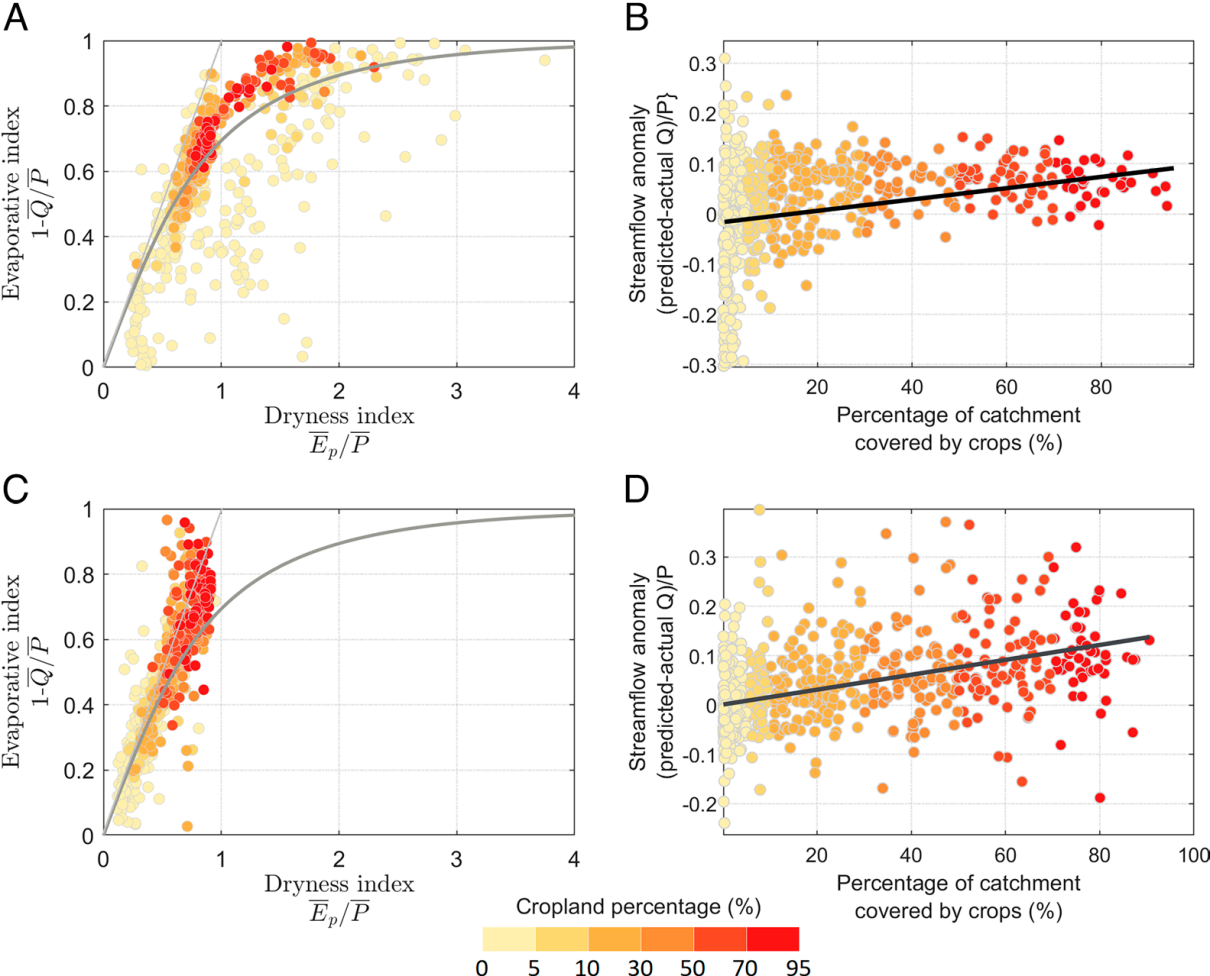
The long-term observed streamflow and precipitation data are analyzed within the framework of the Budyko hypothesis (56). Panels (*A*) and (*B*) depict data for the United States, with (*A*) showing Budyko framework results and (*B*) illustrating the Spearman rank correlation between the percentage of cropland and the anomaly from the Budyko curve. Panels (*C*) and (*D*) present the corresponding results for Great Britain (GB), with (*C*) showing Budyko framework results and (*D*) illustrating the Spearman correlation. The colors indicate the percentage of cropland in each catchment. Spearman ρs between the CL% and the normalized streamflow anomaly in the United States and GB are 0.56 and 0.51, respectively. The normalized streamflow anomaly is calculated as the difference between the simulated (i.e., Budyko-based) and actual streamflow divided by the long-term precipitation (simulated-actual $\bar{Q}$)/$\bar{P}$. These anomalies indicate how agricultural activities alter the natural partitioning of water between evaporation and runoff (See *Materials and Methods* for further information on the equations that were used).

The long-term observations of the 671 CAMELS-US catchments in the context of the Budyko hypothesis are shown in Fig. 2*A*, stratified by the long-term percentage of cropland (CL%). Overall, the observed pattern in the United States aligns with the Budyko curve, with an average exceedance of the normalized mean streamflow ratio (Q/P) by 0.02. Additionally, the analysis reveals that higher CL% corresponds to a higher evaporative fraction (E/P) and decreased runoff ratio (Q/P). The CL% in the US catchments is significantly (*P*-value < 0.05) correlated with the normalized streamflow anomaly, as shown in Fig. 2*B* (Spearman ρ= 0.56), showing that the agricultural expansion is associated with higher streamflow anomalies. Hereafter, when we use the term “significant,” it specifically refers to *P*-value < 0.05. The reduction in variability of anomalies in Fig. 2*B* may be attributed to the moderating influence of agricultural practices in catchments with higher cropland coverage, where hydrological processes are more strongly governed by agricultural activities than by other natural mechanisms.

Fig. 2*C* shows the results for the 671 CAMELS-GB catchments in GB. The pattern of observations deviates further from the Budyko curve compared to the US catchments with a mean exceedance of the normalized Q/P of 0.04. Like the US catchments, the CL% is associated with normalized Q/P anomaly (Spearman ρ =0.51), as shown in Fig. 2*D*. The analyses in both the United States and GB show that agricultural activities contribute to deviations from the Budyko curve.

**Fig. 3.**
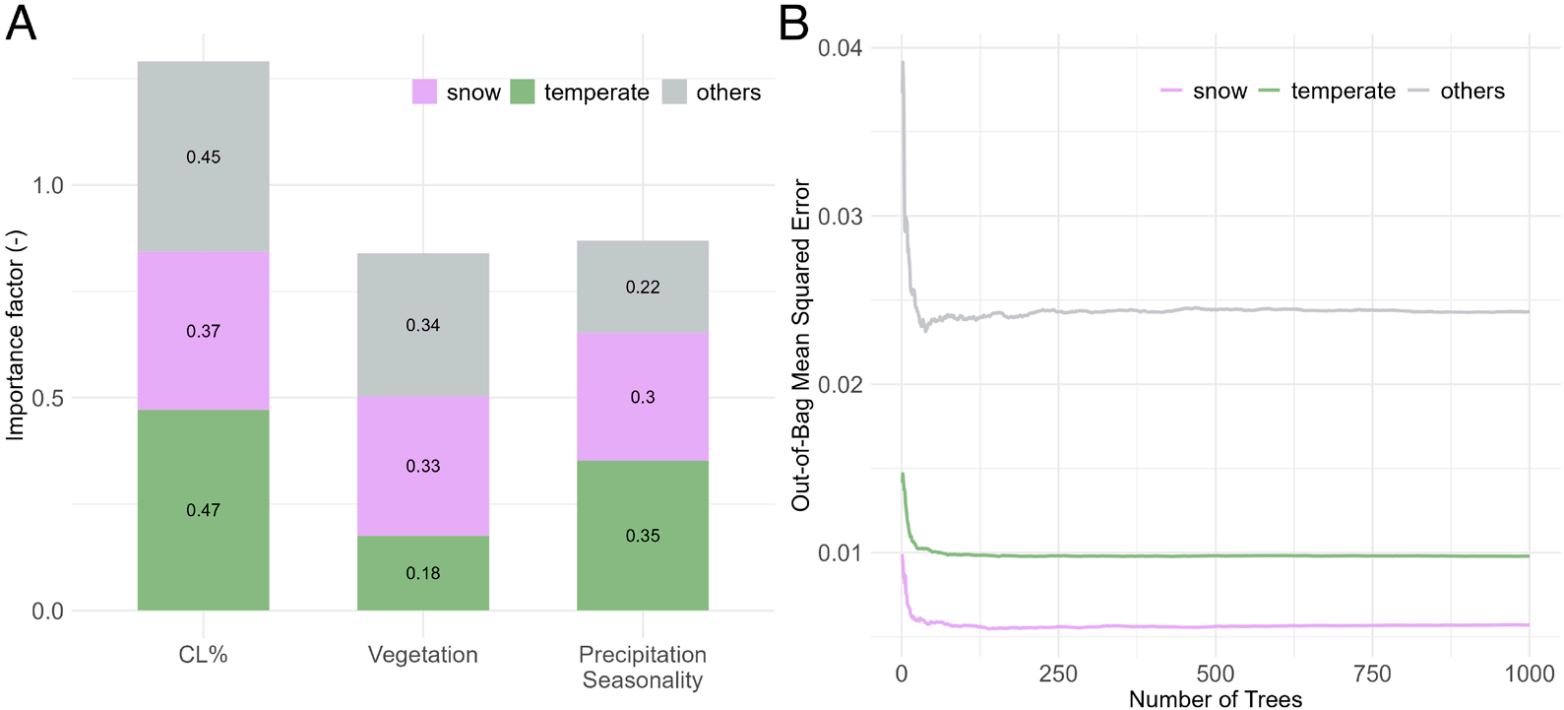
RF model analysis of the relative importance of cropland percentage (CL%), vegetation, and precipitation seasonality in explaining deviations from the Budyko curve for catchments classified by the Köppen-Geiger climate classification. Panel (*A*) presents the factor importance for three climate classes. Panel (*B*) shows the OOB Error against the number of trees for each class. The analysis highlights that CL% consistently demonstrates the highest importance in temperate and snowy climates, surpassing the influence of vegetation and precipitation seasonality.

However, spatial variations in the water balance can also be influenced by other factors, such as vegetation dynamics (61, 63, 76) and precipitation seasonality (62, 64). To investigate how these factors, alongside agricultural activities, contribute to deviations from the Budyko curve, we conducted a Random Forest (RF) analysis across hydrologically significant divisions, specifically three Köppen-Geiger climate classes: temperate, snow, and others—Please see *SI Appendix*, Fig. S2. In the analysis by climate class, temperate and snowy catchments—which together constitute over 90% of the stations (75.6% temperate and 15.2% snowy)—dominate the findings. These catchments are particularly relevant as they represent the majority of agricultural catchments in the studied regions. Conversely, the others have far fewer stations (9.2%), making their contribution to the overall patterns less significant. Fig. 3 presents the RF analysis, highlighting the contributions of cropland percentage (CL%), vegetation, and precipitation seasonality to deviations from the Budyko curve. The RF models were run with 1,000 trees, with the Out-of-Bag (OOB) error stabilizing after approximately 400 trees in all cases.

The analysis shows that CL% dominates vegetation and precipitation seasonality in its influence. In the temperate climate, CL% demonstrates the highest importance (importance score = 0.47), notably exceeding the contributions of vegetation (importance score = 0.18) and precipitation seasonality (importance score = 0.35). In snow-dominated climates, CL% (importance score = 0.37) remains the most influential, although vegetation (importance score = 0.33) and precipitation seasonality (importance score = 0.3) show relatively higher contributions compared to temperate regions. In the others, which comprise less than 10% of the studied catchments, CL% (importance score = 0.45) again exhibits the greatest influence compared to vegetation (importance score = 0.34) and precipitation seasonality (importance score = 0.22).

Overall, CL% exhibits the highest importance (normalized importance score = 0.43), significantly exceeding the importance of vegetation (normalized importance score = 0.28) and precipitation seasonality (normalized importance score = 0.29). These results are consistent with the analysis presented in *SI Appendix*, Fig. S3 for US and GB catchments, where CL% emerged as the most dominant factor, reinforcing the significant role of agricultural activities in driving hydrological deviations across diverse climatic settings. While vegetation and precipitation seasonality are important, CL% stands out as the primary driver, particularly in temperate climate.

### 2.2. Causal Relationships Between Climatic Drivers and Streamflow Suggest Different Impacts of Agricultural Practices Across Diverse Climatic Settings.

**Fig. 4.**
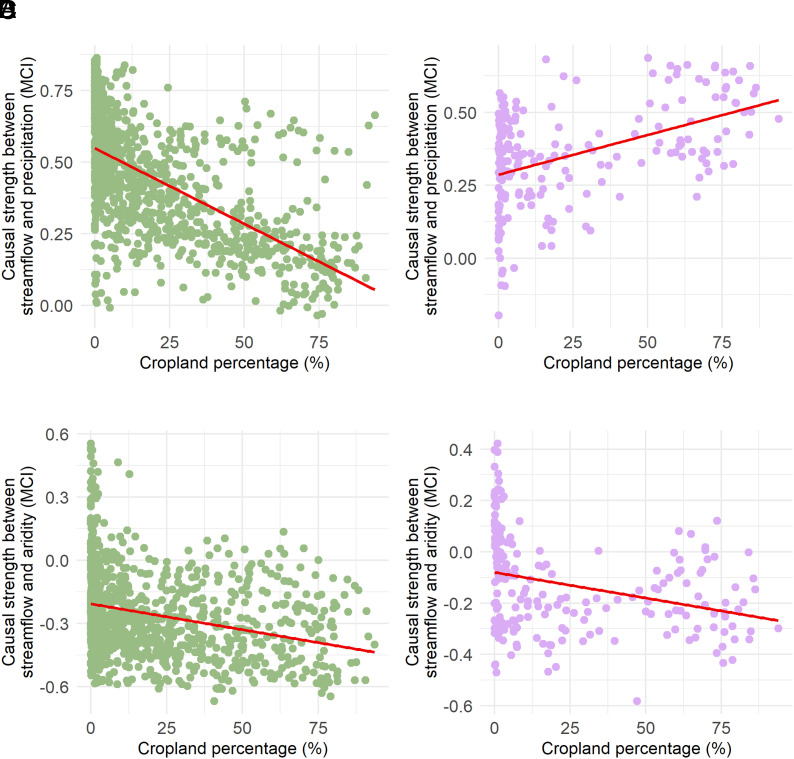
The scatter plots illustrate the correlation between changes in the causal link for P-Q, AR-Q, and cropland percentages (CL%) across temperate and snowy catchments. Panels (*A*) and (*B*) present the P-Q results for temperate and snowy catchments, respectively. Temperate catchments exhibit a strong negative correlation between CL% and P-Q causal strength (Spearman ρ = −0.75), suggesting that cropland exacerbates precipitation-driven deviations from the Budyko curve. Conversely, snowy catchments show a positive strong dependence on precipitation in these regions (ρ = 0.51). Panels (*C*) and (*D*) depict AR-Q results for temperate and snowy catchments, respectively. In temperate catchments, a moderate negative correlation (ρ = −0.42) highlights the role of cropland in influencing streamflow through AR-driven evaporative demand. Similarly, snowy catchments show a negative correlation (ρ = −0.45), underscoring AR’s role as a secondary driver. These results reinforce the varying impacts of agricultural intensity on the water–energy balance components across climatic conditions.

To specifically investigate the influence of agriculture on the water–energy balance, hereafter we focus on the connection between CL% and the causal relationship between observed P-Q and AR-Q across climate classes as shown in Fig. 4 and the two countries in *SI Appendix*, Fig. S4. This dual focus provides insights into how agricultural intensity interacts with water–energy balance components to shape hydrological behavior over different climates and regions.

For temperate catchments, we found a strong negative correlation between CL% and P-Q causal strength (Spearman ρ = −0.75), highlighting the role of cropland in exacerbating precipitation-driven deviations from the Budyko curve. In contrast, the relationship between CL% and AR-Q causality in temperate catchments was weaker but still significant (ρ = −0.42), suggesting that cropland influences water–energy balance through complex interactions with precipitation and AR. However, snowy catchments exhibited a positive correlation between CL% and P-Q causality (ρ = 0.51), indicating a stronger dependence on precipitation. Snowy catchments showed a negative correlation between CL% and AR-Q causal strength (ρ = −0.45), reinforcing the role of AR as a secondary driver. Fig. 4 further demonstrates that the relationships between P-Q and AR-Q are not independent but rather vary in their interactions across climate regions and external factors.

The analysis across countries in *SI Appendix*, Fig. S4 supports these class-specific findings while revealing regional nuances. In the United States where stations span a wide range of climates, the CL% was significantly negatively associated with AR-Q causal strength (Spearman ρ = −0.53), consistent with the patterns observed in temperate catchments. However, the relationship between CL% and P-Q causality in the United States was weak (ρ  = 0.12), reflecting the variability in hydrological responses across diverse climates. In GB, a contrasting pattern emerged: CL% had a strong negative correlation with P-Q causality (Spearman ρ = −0.89), suggesting an increased reliance on precipitation for streamflow in agricultural regions. The relationship between CL% and AR-Q causality in GB was weakly negative (ρ = −0.26), aligning with its temperate climate, where AR plays a less dominant role in water–energy interactions.

### 2.3. Causal Relationships Suggest That Agriculture in the Temperate and Snow Catchments Relies on Different Water Sources.

The preceding analysis implies different impacts of agriculture on the interplay among streamflow, precipitation, and AR. In temperate catchments, causal analysis reveals a direct impact of agriculture on P-Q relationships, indicating that the P-Q causal relationship weakens as CL% increases in a catchment. That is, in crop catchments, P tends to exert a lower influence on Q. However, this is not observed in snowy catchments, where the impact of agriculture on the water balance differs significantly. To further investigate the sources of water for agriculture particularly in the snowy catchments, we partition streamflow into two components: direct flow (Qd; immediate runoff-fed component) and baseflow [Qb; groundwater-fed component (77, 78) utilizing a one-parameter low-pass filter (79–84) (See *Materials and Methods* for details). We examine the relationship between the rate of change in the annual baseflow and CL% to better understand the impact of CL% on the changes in the groundwater-fed component of streamflow (i.e., baseflow). We calculate the linear regression trend of normalized annual baseflows (TNB) over the study period and examine its relationship with the percentage of crop cover in the agricultural catchments.

### 2.4. Agricultural Catchments in a Snowy Climate in the United States Experience Decreases in Baseflow, Suggesting That Streams Are Increasingly Disconnected from Groundwater.

**Fig. 5.**
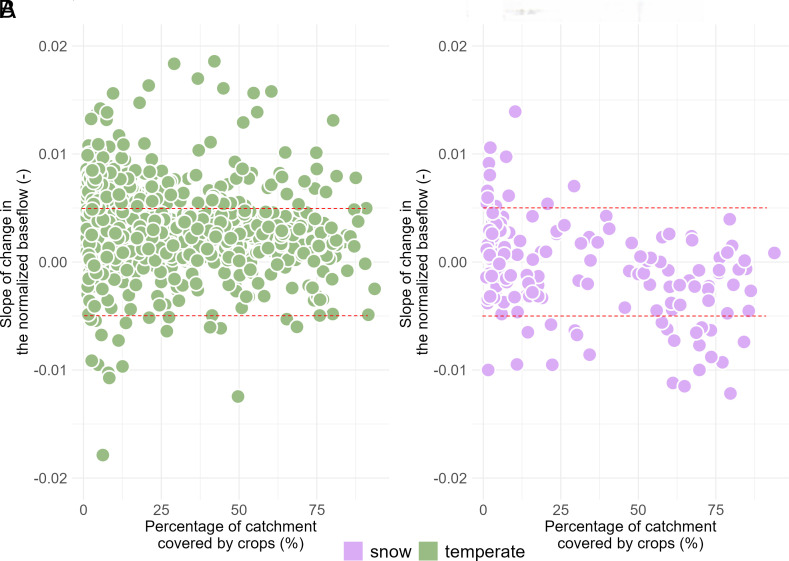
Analysis of normalized baseflow changes in catchments across the temperature and snowy catchments, where cropland constitutes at least 1% of the total basin area, as depicted in Panels (*A*) and (*B*), respectively. Dashed lines represent the significance level (i.e., the threshold for regression trend) with a *P*-value of 0.05. The *y*-axis depicts the linear regression trend of normalized baseflow, while the *x*-axis represents the percentage of the catchment covered by crops. In the snowy catchments, the baseflow’s regression trend strongly correlates with CL%, indicated by a Spearman correlation coefficient of −0.48. In temperature catchments, conversely, the linear regression trend in baseflow demonstrates a low correlation with CL% (Spearman *ρ* = −0.19).

Fig. 5 summarizes findings of regression analysis of TNB, with the y-axis representing TNBs and the x-axis denoting CL%. Panels (*A*) and (*B*) display the results for the temperate and snowy catchments, respectively. The TNBs are determined through a simple linear regression trend, with dashed lines indicating the significance level (i.e., regression trend) at *P*-value = 0.05.

In the snowy catchments, a significant association is observed between the rate of baseflow change and crop cover, as evidenced by a Spearman rank correlation coefficient of −0.48 (95% CIs spanning from −0.55 to −0.41). Remarkably, 38% of snowy catchments exhibit negative trends in baseflow, and among them, 10.3% display statistically significant changes. In contrast, the relationship between TNB and crop cover in temperate catchments appears comparatively weak, indicated by a Spearman correlation coefficient of −0.19. The 95% CI extends from −0.29 to −0.1, underscoring the modest nature of this association. This difference can be attributed to the reliable availability of precipitation in temperate climates, making agriculture primarily dependent on precipitation rather than groundwater. Consequently, the correlation between baseflow changes and CL% is not as pronounced as it is in snowy catchments. In temperate catchments, 13.6% of agricultural catchments demonstrate negative trends in baseflow, and among them, a mere 2.3% exhibit statistically significant changes unrelated to agricultural practices.

## 3. Discussion

The future of terrestrial freshwater ecosystems is uncertain in an era of human interventions and rapid global climate change (85–87). Although previous methods employed modeled streamflow data to identify causal connections between human-induced forcing and observed river flow patterns globally (88–90), uncertainties remain regarding the complex interactions between human activity, climate variables, and catchment-scale hydrological processes that impact water flow dynamics. These uncertainties increase the likelihood of unexpected outcomes as catchment modifications continue at the global scale, including urbanization (91, 92), increased water use (93), and alterations in the structure of land cover such as agricultural expansions (94, 95). Building on previous findings, we have used a large, measured dataset to better understand watershed responses to changes in land surface characteristics caused by agricultural expansion.

To advance our understanding of the water–energy balance and its association with human activities, it is imperative to identify driving forces. Earlier research primarily utilized the Budyko framework to evaluate the sensitivity of streamflow to certain variables. However, this framework, while effective in explaining spatial differences in streamflow across natural catchments, proves less adept at elucidating temporal changes within catchments, particularly concerning climate and human intervention. The intrinsic space-time asymmetry of the Budyko framework raises questions about its suitability for exploring water availability sensitivity at finer temporal scales (68, 69). To address this, we use empirical data and the PCMCI+ causal discovery method (57, 58) to shed light on how agriculture affects the long-term water–energy balance.

Our findings underscore that agricultural expansions induce alterations to the water–energy balance in catchments, contributing to deviations from the Budyko curve. Fig. 4 shows a significant association between agricultural expansion and the AR-Q causal relationship within these areas, indicating that more crop cover exacerbates the negative link between streamflow and AR in these areas. In catchments that rely primarily on groundwater for their water supply, this phenomenon is linked to increased crop cover, which increases evapotranspiration and, in turn, reduces surface runoff and streamflow (96–99) (Fig. 4). This finding is consistent with previous research that highlights the significant effects of groundwater-fed irrigation on evapotranspiration and alterations in the water–energy balance in the US High Plains (17, 33, 94, 100).

In particular, studies show that groundwater plays a critical role in sustaining agricultural activity in US locations with limited water availability. Groundwater-fed irrigation in the US High Plains increases evapotranspiration, which changes the water–energy balance (100). Other studies showed that groundwater levels are widely declined, especially in regions with extensive crop cover (101, 102). Our research supports these conclusions by showing how groundwater-fed agriculture affects departures from the Budyko water–energy balance, reflecting the need of groundwater to meet the crop evapotranspiration (103, 104). This implies that groundwater gains or losses are more likely to have an impact on the theoretical relationship between dryness and the evaporative index (105, 106). Moreover, evapotranspiration and streamflow in the United States are significantly impacted by groundwater losses (94), with changes in evapotranspiration closely linked to crop development. This highlights the disruptive impact of irrigation expansion on the water–energy balance dynamics (107–109). While our spatial analysis offers valuable insights into the role of agriculture in shaping the water–energy balance, it is important to recognize that factors such as the length of the growing season, crop types, and vegetation replacement (110) can further influence temporal evapotranspiration dynamics, introducing complex interactions that may not be fully captured by our approach. Additionally, cropland greatly affects evapotranspiration and energy fluxes, further altering energy balance (106, 109). However, the P-Q relationship does not show a significant association with CL% in US agricultural catchments. This could be because groundwater is typically used instead of direct precipitation to meet agricultural water needs.

On the other hand, in GB, which is characterized by catchments with limited energy in which precipitation is the main source of water for agriculture, changes in the water–energy balance are primarily characterized by variations in the strength of causal relationships between streamflow and precipitation (P-Q). Results show a significant association between the agricultural expansion in temperate catchments and the P-Q relationship, which is not the case in the snowy catchments. As the percentage of CL% rises, the P-Q relationship decreases, suggesting that higher crop cover may result in a lower streamflow ratio as a consequence of changes in the P-Q dynamics. This phenomenon, which is particularly apparent in areas where agriculture significantly depends on precipitation, can be explained by enhanced evapotranspiration and plant uptake of water (106, 107). The absence of correlation between CL% and the AR-Q relationship in temperate catchments can be ascribed to its relatively humid climate, where AR exerts less constraint on streamflow (111, 112). Studies conducted in GB suggest that elevated water usage by crops may result in a decreased distribution of precipitation, favoring evapotranspiration over runoff (111–113), which affects, in turn, the P-Q relationship. The reduction of the discrepancy between actual and potential evapotranspiration is largely dependent on the interplay between soil water storage and crop responses to water availability. This leads to a linear relationship between the evaporative ratio and AR (106, 107, 114) in these areas.

These distinct patterns underscore the nuanced interplay between agriculture and the water–energy balance, further emphasizing the impact of human activities as well as geographical and climatic variations on catchment dynamics. Understanding these intricacies is essential for effective water resource management, especially considering the potential implications of climate change and agricultural practices on streamflow patterns in energy-limited catchments like those in GB.

In conclusion, our study emphasizes the intricate relationship between agriculture by water source, the water–energy balance in a catchment, and deviations from the Budyko curve. A more profound understanding of these dynamics equips us to navigate water resource management adeptly, advocate for sustainable agricultural practices, and progress toward attaining global sustainable development goals. While our primary analysis centered on deviations from the Budyko curve due to alterations in the water–energy balance induced by agricultural practices, it is crucial to acknowledge additional influential factors shaping regional and global hydrological patterns. These factors encompass climatic variables such as climate variability and change (40, 115), human activities like drainage and groundwater extraction for domestic and industrial purposes, as well as catchment characteristics (37, 116, 117). For instance, in the Southeast region of GB, where extensive agricultural activities happen, rainfall-runoff events exhibit low runoff coefficient values. This is mainly due to the presence of chalk catchments characterized by high permeability (118). The occurrence of lateral subsurface flow in these catchments warrants consideration; however, its impact on the arguments presented in this paper is minimal, as the water involved typically travel the saturated zone swiftly and contributes to flow over the course of months (119, 120).

In attributing the change in the hydrological cycle to CL% as a proxy for agriculture, there is an opportunity for further research into the interplay of human and natural hydrological dynamics. For instance, a more comprehensive investigation of P-Q dynamic in GB can provide insights into the relationship between changes in the water–energy balance, CL%, and the implementation of field drainage in the 1970s and early 1980s (117). This, in turn, can unveil how agricultural practices aimed at reducing waterlogged conditions have impacted crop yield, transpiration, and water storage capacity within the upper soil profile, leading to changes in runoff changes (121–123). These multifaceted interactions warrant continued exploration and consideration in our ongoing efforts to address water-related challenges and secure a more sustainable future.

## 4. Materials and Methods

### 4.1. Data.

The research focuses on analyzing watershed data from two comprehensive datasets, one from the United States and the other from GB, to understand hydrological variations across these regions. The US data are derived from the CAMELS dataset (124), which includes detailed information on 671 catchments across the contiguous United States. These catchments vary significantly in size, from 4 to 25,817 square kilometers, and provide a rich source of data including discharge, meteorological information, and various other attributes from 1980 to 2015. Key variables such as precipitation, temperature, potential evaporation, and streamflow are included, with streamflow data specifically sourced from the HCDN-2009 network (125). Additionally, the dataset incorporates information on cropland extent, calculated using a global cropland map (126), and groundwater-level data from the US Geological Survey (https://waterdata.usgs.gov/nwis/inventory).

**Fig. 6.**
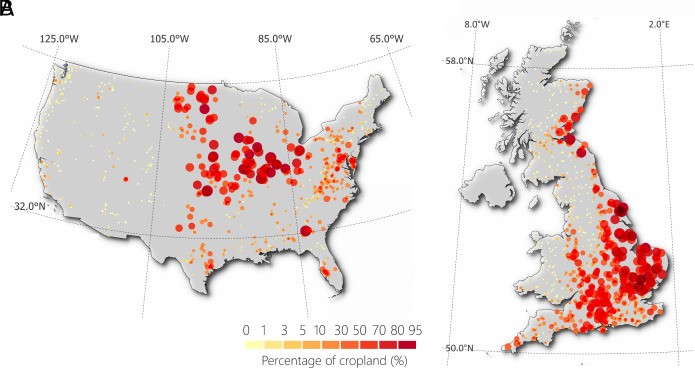
Geographical distribution of studied catchments. Panels (*A*) and (*B*) display the locations of CAMELS catchments in the United States and GB, respectively. The shades of red color represent the percentage of cropland. The size of dots is proportional to the magnitude of the percentage of cropland.

In contrast, CAMELS-GB dataset (127), encompasses hydrometeorological and attribute data for 671 catchments across GB. These catchments also display a wide range of sizes, from 1.6 to 9,930 square kilometers, and include daily time series data for variables such as temperature, precipitation, potential evapotranspiration, and streamflow, covering the period from 1970 to 2015. The creation of the CAMELS-GB dataset aimed to synthesize existing data into a consistent, up-to-date collection that facilitates the comparability and reproducibility of hydrological studies across GB. This dataset represents a significant effort to provide a comprehensive view of the hydrological and meteorological characteristics of GB catchments, including aspects such as land cover, with crops and grassland being predominant in different regions, and the distribution of large reservoir capacities, especially in the more mountainous areas. Fig. 6 shows the spatial distribution of CAMELS catchments across the United States and GB, stratified with CL%.

Both datasets cover a wide range of climatic, hydrological, landscape, and human management characteristics, underscores the complexity of watershed dynamics and the importance of large-sample studies in advancing our understanding of hydrological systems.

They serve as invaluable resources for researchers aiming to analyze regional variability in hydrologic model performance, assess the impact of climatic and land-use changes on water resources, and develop strategies for sustainable water management.

### 4.2. Snow Estimation.

To employ the causal discovery algorithm and enhance our comprehension of the influences on water balance, it is imperative to pinpoint the primary drivers shaping variations in streamflow. Within this framework, our focus centers on three pivotal factors impacting streamflow (Q): precipitation (P), AR, and SF. Directly extracted from the CAMELS datasets, precipitation and AR data serve as foundational components. For the determination of the proportion of precipitation designated as snow (SF), a straightforward temperature-based threshold was applied. Specifically, when the average daily temperature dipped below 1 °C, all precipitation was categorized as snowfall (66, 128). Conversely, if temperatures surpassed 1 °C, the precipitation was classified as rainfall. Additionally, an alternative threshold of 0 °C (70) was explored, yielding consistent outcomes regardless of the chosen method for estimating snowfall.

### 4.3. Budyko Framework.

Here, we employ the Budyko framework, a well-known approach to examine the complex relationships between streamflow elements, meteorological conditions, and catchment features in relation to the long-term water balance (41, 52, 73). Based on AR index (ϕ), this framework formalizes the partitioning of precipitation (P) into evapotranspiration (ET) and runoff (Q). The framework aids to evaluate the main variables affecting the components of water balance. Through an examination of the ways in which catchments with certain characteristics deviate from the Budyko curve, we may assess how much and how these features affect the components of the water balance beyond the AR index (66, 68). The Budyko curve is represented by the following equation (56):

[1] $$\frac{1-\bar{Q}/\bar{P}}{\bar{P}}=\sqrt{\frac{{\bar{E}}_{p}}{\bar{P}}\tanh\left(\frac{\bar{P}}{{\bar{E}}_{p}}\right)\left(1-\exp\left(-\frac{{\bar{E}}_{p}}{\bar{P}}\right)\right)},$$

where $\bar{Q}$, $\bar{P}$, and ${\bar{E}}_{p}$ denote the long-term mean values for streamflow, precipitation, and potential evaporation.

### 4.4. RF for Analysis of Attribution.

To identify the factors contributing to deviations from the Budyko curve, it is essential to quantify the relative importance of each variable. Traditional approaches, such as linear or logistic regression models, can be used to evaluate the influence of explanatory variables on a response variable (129). However, multiple regression method is only applicable when there is a linear relationship between predictors and the response variable, and when the assumptions of normally distributed residuals, homoscedasticity, and independence are met (130).

Given the nonlinear interactions between deviations from the Budyko curve and contributing factors, we employed the RF algorithm. RF is well suited for handling complex, nonlinear relationships and interactions, offering robust performance in high-dimensional settings which enables the relative importance of influencing factors (131, 132). It uses an ensemble of Classification and Regression Trees (CARTs), enhancing predictive accuracy and reducing variance compared to single decision trees (133). The model employs bootstrap aggregation (“bagging”) to create multiple subsets of the original data via random resampling with replacement, allowing each tree to train on unique subsets. Data points not selected in these samples form “OOB” samples, which are critical for assessing variable importance.

To evaluate the importance of a variable, RF permutes the variable in the OOB samples while keeping others constant and reruns the model. The variable’s importance is calculated as the average difference in prediction accuracy between the original and permuted OOB samples. Mathematically, the variable importance (VI) of an explanatory variable x is defined as

[2] $$\mathrm{VI}\left(x\right)=\frac{1}{\mathrm{ntree}}\sum_{i=1}^{\mathrm{ntree}}\left({\tilde{y}}_{i}-y_{i}\right),$$

where y is the prediction with the original OOB sample, ${\tilde{y}}_{i}$ is the prediction with the permuted OOB sample, and ntree denotes the total number of trees in the forest. A larger value of VI indicates a higher importance of the variable.

In this study, RF was used to evaluate the relative importance of cropland percentage (CL%), vegetation, and precipitation seasonality in explaining deviations from the Budyko curve, offering insights into the key drivers of hydrological behavior across different catchments.

### 4.5. Causal Discovery Algorithm.

In our study, we aim to understand the impact of agriculture on the water cycle across various catchments in the United States and GB by examining causal relationships between climate variables and streamflow. Traditional approaches, like the Budyko hypothesis, provide valuable insights into the spatial influence of agriculture on the water cycle; however, they fall short in explaining the causal mechanisms behind observed deviations in water balance. Most statistical attribution studies rely on qualitative reasoning, sensitivity-based analysis, or correlation-based techniques, which often focus on a single driver and do not adequately quantify causal relationships among multiple interacting factors (66, 69, 134, 135).

**Fig. 7.**
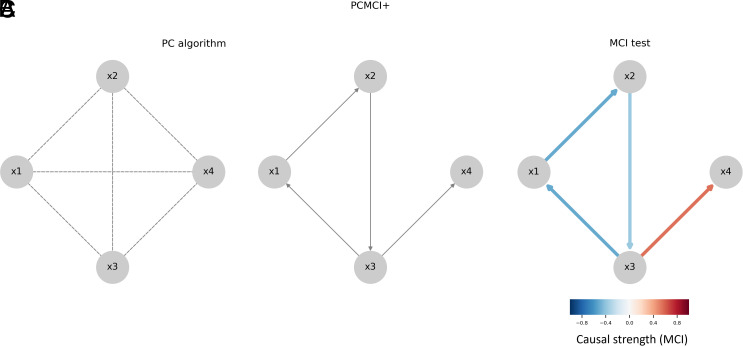
Overview of the PCMCI+ causal discovery algorithm. The algorithm consists of two main stages: the PC algorithm and MCI tests. In the first stage (Panel *A*), the PC1 algorithm begins with a fully connected graph. It then iteratively removes connections between variables by testing conditional independence with sets of increasing size (Panel *B*). In the second stage (Panel *C*), the MCI tests apply the conditions identified in the first step to determine strength of causal links.

To address these limitations, we employ a causal discovery approach using the Peter-Clark MCI Plus (PCMCI+) algorithm. This algorithm allows us to explore the temporal interactions among hydrological components that shape deviations from the Budyko curve, without violating the principle of mass conservation (P - ET = Q). The focus is on understanding how specific drivers, such as SF or cropland percentage (CL%), perturb these relationships over time, leading to deviations in long-term averages. PCMCI+ is specifically designed to detect both contemporaneous (simultaneous) and time-lagged causal relationships in high-dimensional time series data, which is common in environmental studies (136, 137). The PCMCI+ algorithm identifies causal connections among variables. It starts by constructing a Directed Acyclic Graph (DAG), which represents the system’s causal structure (58, 138). In a DAG, variables are represented as nodes, and directed edges (arrows) illustrate causal relationships between them as shown in Fig. 7.

The PCMCI+ algorithm operates in two main stages (139). The first stage, known as the PC Stage (Conditional Independence Testing), uses the PC1 algorithm, a variant of the original Peter-Clark (PC) algorithm, to identify a set of variables that best explains the dependencies of that variable. This stage begins with a fully connected graph as shown in Fig. 7*A* and tests conditional independence between variables by incrementally increasing the number of variables in the conditioning set. By applying linear partial correlation tests, the algorithm determines which connections between variables can be removed while preserving the graph’s validity. The process continues iteratively, aiming to converge on a subset of essential links at a significance level of 0.05, ensuring that only significant causal links remain as shown in Fig. 7*B*.

In the second stage, known as the MCI Stage (Causal Inference), the MCI test leverages the estimated conditions identified during the PC stage to infer causal relationships between variables. This stage assigns a causal strength value, measured by MCI partial correlation, to each detected causal link (74, 75) (Fig. 7*C*). The MCI test can capture both linear and nonlinear dependencies, providing a deeper understanding of causal interactions. Together, these two stages allow the PCMCI+ algorithm to effectively distinguish direct causal influences from indirect or spurious connections, enhancing the reliability of the resulting causal graph. The Python software for estimating the causal network is available at https://jakobrunge.github.io/tigramite.

### 4.6. Decomposition of Streamflow.

To further explore the analysis of water sources for agriculture, we utilized a one-parameter low-pass filter method developed by Lyne and Hollick (79–84), commonly used in the literature (82, 84, 140, 141) to effectively decompose streamflow into its constituent components, namely baseflow and direct flow. This method relies on temporal filtering principles, wherein the streamflow hydrograph undergoes convolution with a smoothing function defined by a filter parameter, often referred to as the recession constant (α). We applied the recursive filter iteratively three times (forward-back-forward) to filter off flood peaks from the original streamflow time series, configuring the filter parameter [set as 0.925; a widely accepted value in hydrological studies (79, 80, 140, 142–144). The resulting separation let us to discern the slow, groundwater-derived baseflow from the rapid, rainfall-induced direct flow. To account for the inherent uncertainty of streamflow partitioning (81), we repeated our analysis using the Lyne Holick one-pass configuration, which yielded quite similar results.

To calculate baseflow, we used Lyne and Hollick’s (79–84) one-parameter low-pass filter approach, which is a well-known method in the literature (82, 84, 140, 141). By applying temporal filtering principles, this approach effectively partitions streamflow into its two main components: baseflow and direct flow. Recession constant (α), a filter parameter, is used to build a smoothing function that is used to convolve the streamflow hydrograph. We applied the recursive filter three times (forward-back-forward), using a filter parameter set to 0.925, a widely accepted value in hydrological studies (79, 80, 140, 142–144). The original streamflow time series’ flood peaks were effectively removed by this iterative procedure, making it possible to distinguish between rapid, rainfall-induced direct flow and slow, groundwater-derived baseflow. To account for the inherent uncertainty of streamflow partitioning (81), we repeated our analysis using the Lyne Holick one-pass configuration, which led to similar results.

## Supplementary Material

Appendix 01 (PDF)

## Data Availability

Catchments in Great Britain (CAMELS-GB), daily hydro-meteorological time series and landscape features; data for the US (CAMELS); causal network estimation code data have been deposited in Environmental Information Data Centre; Geoscience Data Exchange Centre of the NSF; Potsdam Institute for Climate Impact Research (PIK) (https://catalogue.ceh.ac.uk/documents/8344e4f3-d2ea-44f5-8afa-86d2987543a9; https://dx.doi.org/10.5065/D6MW2F4D; and https://tocsy.pik-potsdam.de/tigramite.php). Previously published data were used for this work (124, 127).
