# Supplementary material for: Agriculture’s impact on water–energy balance varies across climates
Source: Proc Natl Acad Sci U S A. 2025 Mar 17;122(12):e2410521122. doi: 10.1073/pnas.2410521122 (PMC11962491; doi:10.1073/pnas.2410521122)
Supplement: Supplementary file 1 — Appendix 01 (PDF) [file pnas.2410521122.sapp.pdf]

Supporting Information for
Agriculture's Impact on Water-Energy Balance Varies Across Climates

Masoud Zaerpour[*,1], Shadi Hatami[1], André S. Ballarin[2], Simon Michael Papalexiou[1,3], Alain Pietroniro[1], Ali Nazemi[5]

[1]Department of Civil Engineering, Schulich School of Engineering, University of Calgary, Canada; [2]Department of Hydraulics and Sanitation, São Carlos School of Engineering, University of São Paulo, Brazil; [3]Faculty of Environmental Sciences, Czech University of Life Sciences, Czech Republic; [4]Department of Building, Civil, and Environmental Engineering, Concordia University, Canada

Masoud Zaerpour
Email: masoud.zaerpour@ucalgary.ca

This PDF file includes:

Figures S1 to S4

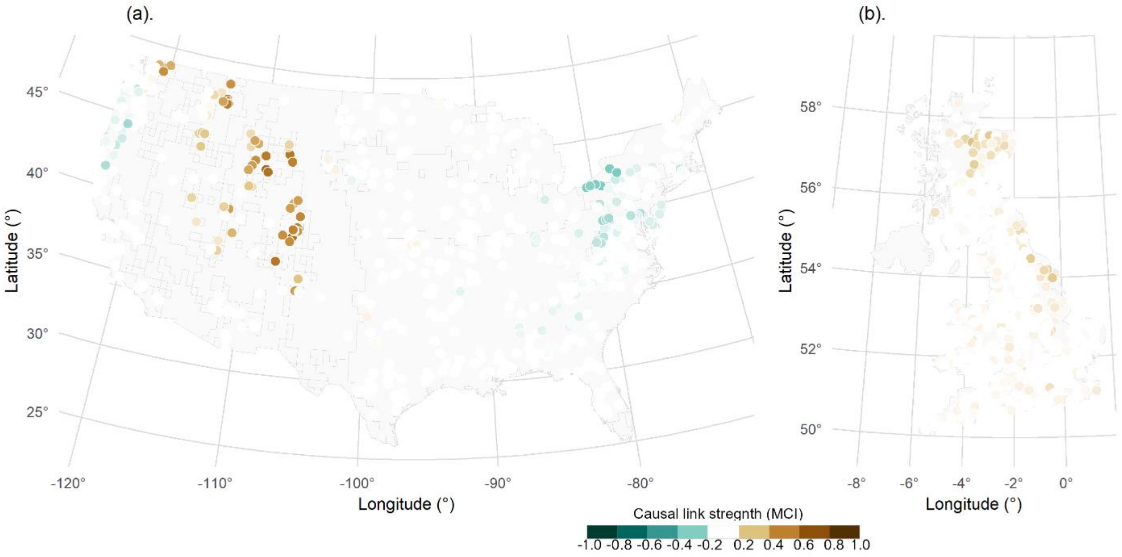

Fig. S1. Quantifying the causal link strength between streamflow and snow fraction. The strength of these causal links is assessed through the MCI test statistic, where MCI values range from -1 to 1. Panels (a) and (b) illustrate the spatial distribution of causal links between streamflow and snow fraction (SF-Q) in the US and GB, respectively. The size of dots is proportional to the magnitude of MCI values.

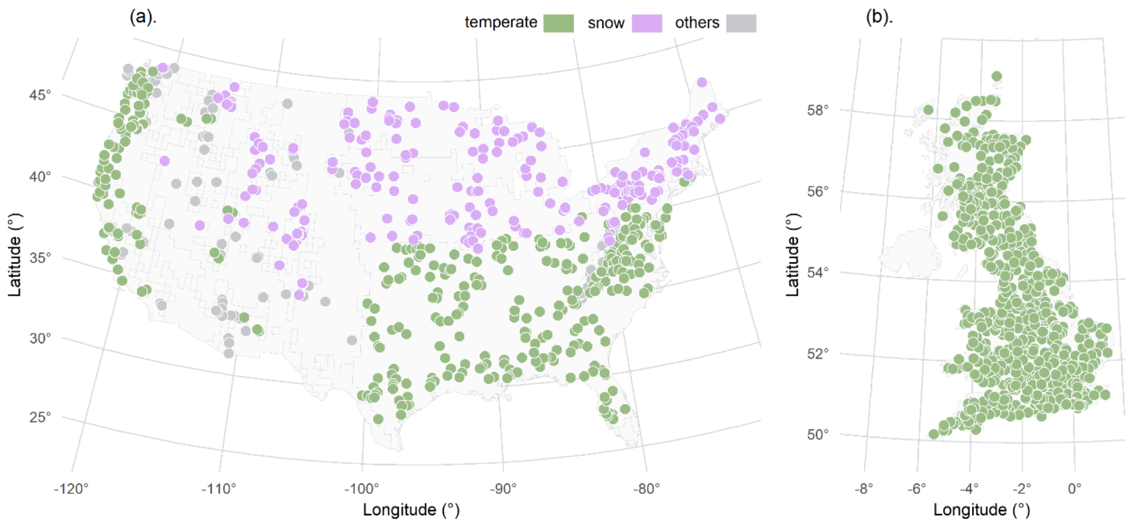

Fig. S2. The spatial distribution of CAMELS stations across the US and GB. Colors show the climate classes each catchment belongs to: temperate (75.6%), snow (15.2%), and others (9.1%).

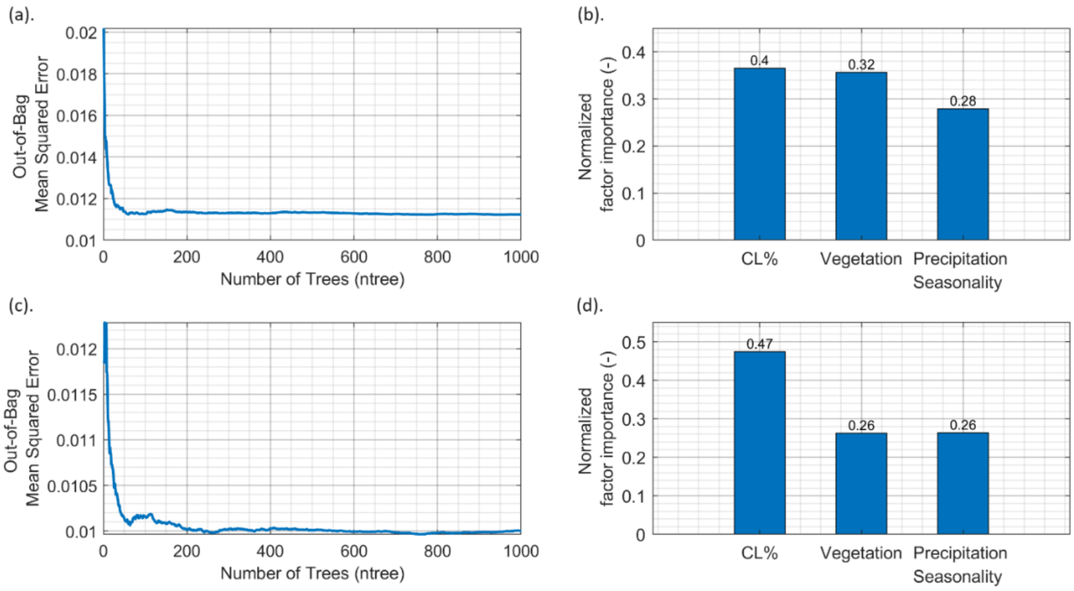

Fig. S3. Random Forest (RF) model analysis of the relative importance of cropland percentage (CL%), vegetation, and precipitation seasonality in explaining deviations from the Budyko curve for US and GB catchments. The top row represents the results for US catchments ($R^2 = 0.78$), showing the Out-of-Bag (OOB) Error against the number of trees and the normalized factor importance. The bottom row depicts the same analysis for GB catchments ($R^2 = 0.63$). The analysis highlights that while vegetation and precipitation seasonality are influential, the contribution of agricultural activities (CL%) is more pronounced, particularly in the GB catchments.

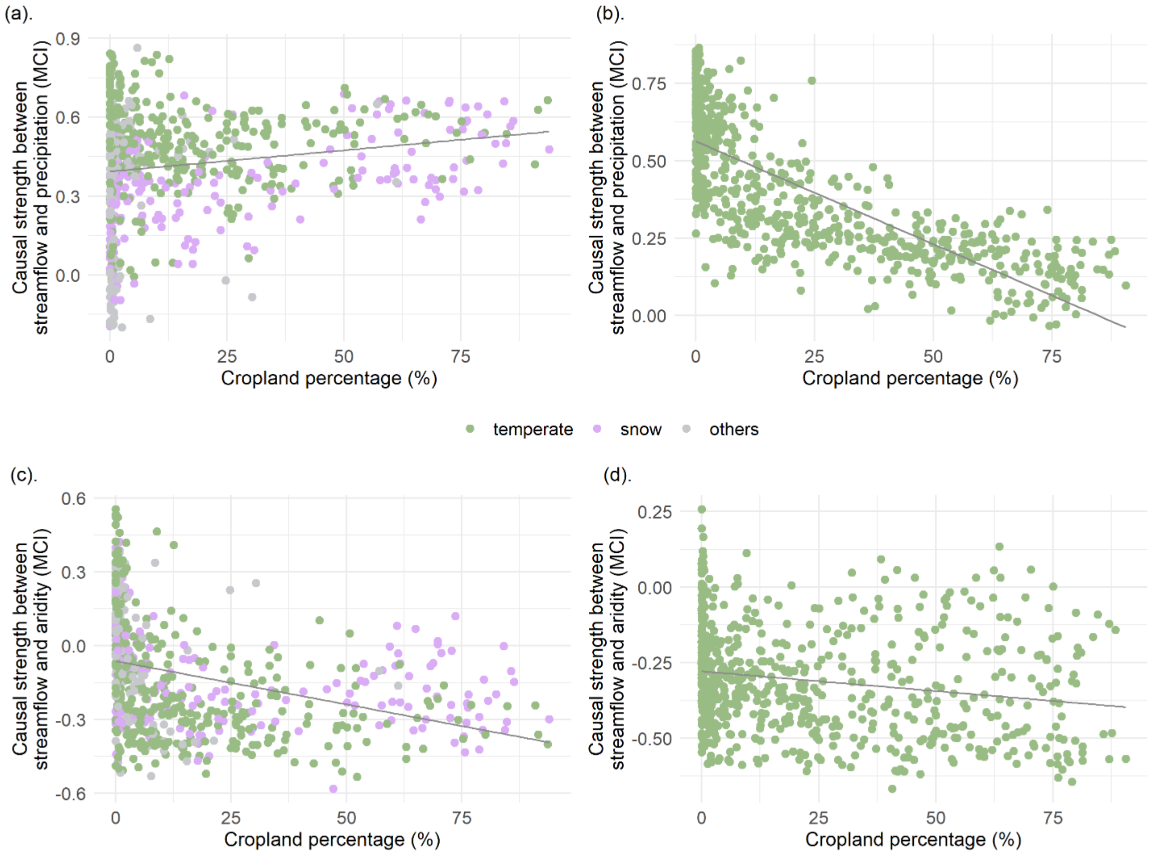

Fig. S4. The scatter plots illustrate the correlation between changes in the causal link for P-Q, AR-Q, and cropland percentages (CL%). Panels (a) and (b) correspond to P-Q results in the US and GB, respectively. In the US, panel (a) indicates a weak correlation between CL% and P-Q causal strengths, revealing a consistent line around 0.4, highlighting the relationship between precipitation and streamflow. Conversely, in GB, panel (b) demonstrates a strong association between CL% and P-Q causal strengths, supported by a Spearman rank correlation coefficient of -0.89 (95% CI: -0.90 to -0.87). Notably, catchments in GB with a higher percentage of crops exhibit a lower causal link between streamflow and precipitation. Panels (c) and (d) showcase results for AR-Q in the US and GB, respectively. In the US, panel (c) reveals a significant association between CL% and changes in AR-Q causal links, with a Spearman rank correlation coefficient of -0.53 (95% CI: -0.56 to -0.51). However, in GB, panel (d) indicates no association between AR-Q causal strengths and CL%.
